# The Relationship Between OCT and VEP Parameters with Disability and Disease Duration in Relapsing–Remitting Multiple Sclerosis

**DOI:** 10.3390/diagnostics15172181

**Published:** 2025-08-28

**Authors:** Manuela Andreea Ciapă, Vlad Constantin Donica, Claudia Florida Costea, Anisia Iuliana Alexa, Alexandra Lori Donica, Camelia Margareta Bogdănici

**Affiliations:** 1 Doctoral School, Faculty of Medicine, University of Medicine and Pharmacy “Grigore T. Popa”, University Street, No. 16, 700115 Iasi, Romania; manuela-andreea_v_tanasachi@d.umfiasi.ro (M.A.C.);; 2 Department of Ophthalmology, Faculty of Medicine, University of Medicine and Pharmacy “Grigore T. Popa”, University Street, No. 16, 700115 Iasi, Romaniacamelia.bogdanici@umfiasi.ro (C.M.B.)

**Keywords:** multiple sclerosis, optical coherence tomography, visual evoked potentials, optic pathway, neurodegeneration, EDSS, optic neuritis

## Abstract

**Background:** Optic neuritis (ON) is a common manifestation of multiple sclerosis (MS), serving as a clinical window into central nervous system demyelination. Optical coherence tomography (OCT) and visual evoked potentials (VEPs) are complementary non-invasive tools for assessing structural and functional damage to the visual pathway. The objective of this paper is to evaluate correlations between OCT and VEP parameters in MS patients with and without a history of ON and assess their relationship with disease duration and disability (EDSS). **Methods:** This cross-sectional study included 54 eyes from 27 relapsing–remitting MS patients. OCT was used to measure circumpapillary and the temporal peripapillary retinal nerve fiber layer (pRNFL) and the foveal/parafoveal ganglion cell-inner plexiform layer (GCIPL) thickness. VEPs assessed P100 latency and amplitude. Patients were grouped by ON history. **Results:** Eyes without ON showed a significantly greater circumpapillary pRNFL thickness (mean difference: 18.27 ± 5.33 µm, *p* = 0.001), temporal pRNFL thickness (15.71 ± 5.49 µm, *p* = 0.006), and parafoveal GCIPL thickness (12.85 ± 5.3 µm, *p* = 0.019) compared to ON eyes. p100 latency was shorter and the amplitude was higher in NON eyes, but without statistical significance. Strong negative correlations were found between OCT thickness and EDSS and disease duration. p100 latency correlated negatively with OCT parameters, while amplitude showed a positive correlation with pRNFL thickness in ON eyes. **Conclusions:** OCT parameters, particularly pRNFL and GCIPL thickness, correlate with functional and clinical markers of MS. Combined OCT–VEP evaluation enhances the assessment of neurodegeneration and disease progression.

## 1. Introduction

Multiple Sclerosis (MS) is a chronic inflammatory demyelinating disease of the central nervous system (CNS) characterized by multifocal areas of demyelination and axonal loss [1]. Among the earliest and most common clinical manifestations of MS is optic neuritis (ON), which represents the inflammation of the optic nerve, patients suffering from a decrease in visual acuity, loss of the visual field, color and contrast sensitivity, and periocular pain [2]. Around 70% of MS patients will develop ON over the course of their disease, while 20% can have it as a first MS symptom [3]. Therefore, analyzing the integrity of the optic nerve in MS patients can provide a unique window into disease pathophysiology due to the accessibility and functional relevance of the visual pathway [4].

While the current McDonald criteria are based on the 2017 revisions and only recommend incorporating the anterior optic pathway as diagnostic criteria [5,6], the 2024 proposed revision suggested adding the optic nerve as a fifth topographic location of the CNS, increasing the value of investigations that could be used to objectify the presence of a demyelinating lesion located across the optic pathway [7]. This aspect was first proposed by The Magnetic Resonance Imaging in Multiple Sclerosis (MAGNIMS) group in order to demonstrate dissemination in space in MS, which may be associated with an earlier and more accurate diagnosis [8].

Visual evoked potentials (VEPs) and optical coherence tomography (OCT) are two complementary, non-invasive tools that enable the assessment of the structural and functional integrity of the anterior visual pathway [9]. VEP provides objective measures of functional conduction delays resulting from demyelination manifested through alterations of the amplitude and latency of the p100 wave complex [10], while OCT allows high-resolution quantification of retinal axonal and neuronal loss, particularly of the peripapilar retinal nerve fiber layer (pRNFL) and the ganglion cell-inner plexiform layer (GCIPL) [11].

By analyzing posterior segment OCT in MS, we can obtain data regarding the integrity of the optic nerve axons. While the investigation can objectify optic disc alterations in juxtabulbare ON by an increase in pRNFL thickness, it is better suited to provide evidence regarding transsynaptic degeneration after the inflammatory episode occurred through a decrease in both the pRNFL and GCIPL thickness [12]. Using VEPs, information regarding the integrity of the entire visual pathway is obtained, which may improve the detection of ON and an evaluation of recovery after the resolution of the inflammatory episode [13]. Therefore, VEPs may be better suited for evaluating ON onset and resolution, while OCT offers insight regarding neurodegeneration.

Together, these modalities offer valuable biomarkers for detecting subclinical damage, monitoring disease progression, and evaluating therapeutic responses. Their combined use enhances our understanding of neurodegeneration and remyelination processes in MS and contributes to a more comprehensive evaluation of the disease burden beyond conventional MRI [14]. In addition, affection of the optic nerve without other explanation identified using these methods may be used to highlight dissemination in space, converting patients with clinically isolated syndrome to MS [15].

Prior studies have demonstrated varying degrees of correlation between OCT-derived structural parameters and VEP functional metrics, with stronger associations often observed in eyes affected by ON. Furthermore, both OCT and VEP findings have been linked to clinical measures of disability, such as the Expanded Disability Status Scale (EDSS), underscoring their potential utility as biomarkers of disease progression. However, discrepancies in the strength and consistency of these correlations across different cohorts suggest that factors including disease duration, MS subtype, and methodological variability may influence outcomes.

This paper aims to investigate the correlation between structural changes detected by OCT and functional alterations captured by VEPs in patients with MS, with or without a history of ON, and their relationship with EDSS and disease duration, exploring their utility as potential biomarkers in clinical and research settings.

## 2. Materials and Methods

The study design and protocol were conducted in accordance with the tenets of the Declaration of Helsinki for research involving human subjects and approved by the Ethics Committee of “Grigore T. Popa” University of Medicine and Pharmacy Iasi, Romania (No. 203/approval date on 3 July 2022). Written informed consent was obtained prior to patient evaluation.

We performed a cross-sectional analysis of 54 eyes from 27 relapsing–remitting MS patients, analyzing the circumpapillary and temporal pRNFL and foveal and parafoveal GCIPL thickness using OCT, the p100 wave amplitude and latency using VEPs, and EDSS and disease duration parameters. Patients were recruited during their periodic neurological assessment between January 2023 and January 2025. The inclusion criteria were a confirmed RRMS diagnosis, no inflammatory episode within the last three months, and a clinically confirmed ON history for the ON group. The exclusion criteria were composed of other coexisting optic neuropathies, ophthalmological diseases, a recent episode of ON, an inability to cooperate with OCT or VEP examinations, the use of medication with retinal toxicity or systemic conditions with ocular affliction, and the refusal of the patient to participate in the study.

Macular and optic disc-centered OCT were obtained by an experienced examiner using the TRITON swept-source OCT device (DRI OCT Triton, Topcon, Tokyo, Japan), ensuring the same light settings and parameters. The OCT disc scans were reviewed to ensure correct segmentation and high image quality. Layer segmentation and measurements were performed automatically by the integrated Triton software.

The VEP recordings were performed using a NIHON KOHDEN device, with a checkerboard-patterned stimulation, adhering to international recommendations: monocular stimulation, a stimulus rate of 1 cycle/second, a contrast of 0.5 (corresponding to a minimum 3:1 ratio between maximum and minimum illumination), and a visual angle (defined as the size of a single bright or dark element) of 10 min. The amplifier filters were set to 1–30 Hz. The recorded parameters included the p100 wave latency (measured in milliseconds) and the p100 wave amplitude (measured as the peak-to-peak value between the N75 and p100 waves, in microvolts).

Disease duration was collected from the patient’s medical charts, and EDSS was calculated by an experienced neurologist.

### Statistical Analysis

Statistical analysis was performed using the SPSS statistical package, version 26.0. Normality was evaluated using the Shapiro–Wilk test. Normality was not respected for the pRNFL and foveal GCIPL thickness, EDSS, and disease duration in both ON and NON eyes, and for the p100 wave latency in NON eyes. The other parameters presented a normal distribution of normality. An independent-samples *t*-test was used to determine whether there was a statistically significant mean difference between the OCT parameters, EDSS score, disease duration, and the p100 wave latency and amplitude between eyes with a positive history of ON and without. Levene’s test for equality of variances assumed equal variances (*p* > 0.05). The data are the mean ± standard deviation, unless otherwise stated. Outliers were detected that were more than 1.5 box-lengths from the edge of the box in a boxplot. An inspection of their values did not reveal them to be extreme, and they were kept in the analysis. A non-parametric Spearman correlation was run to evaluate the relationship between the investigated parameters in ON, respectively, NON eyes. A level of *p* < 0.05 was accepted as statistically significant.

## 3. Results

We analyzed fifty-four eyes from twenty-seven patients in order to observe existing correlations between the p100 wave latency and amplitude, circumpapillary and temporal pRNFL, foveal and parafoveal GCIPL thickness, disease duration, and EDSS dependent on ON history. Twenty-two eyes presented no history of ON, while thirty-two eyes presented at least one ON episode.

The mean age was 39.08 ± 13.14 years with a female–male ratio of 2:1. Disease duration had a median of 6 ± 9.96 years, while EDSS had a median value of 2 ± 1.4. Descriptive parameters regarding the circumpapillary and temporal pRNFL thickness, the foveal and parafoveal GCIPL thickness, and the p100 wave parameters can be seen in Table 1.

**Figure 1:**
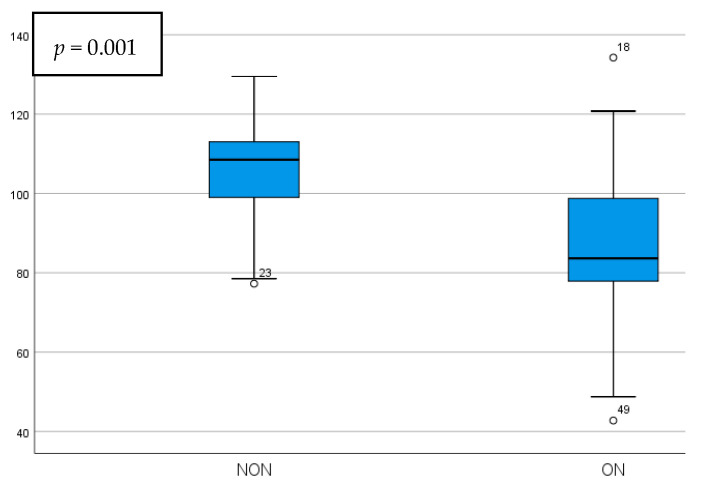
Circumpapillary pRNFL thickness comparison in NON vs. ON eyes.

**Figure 2:**
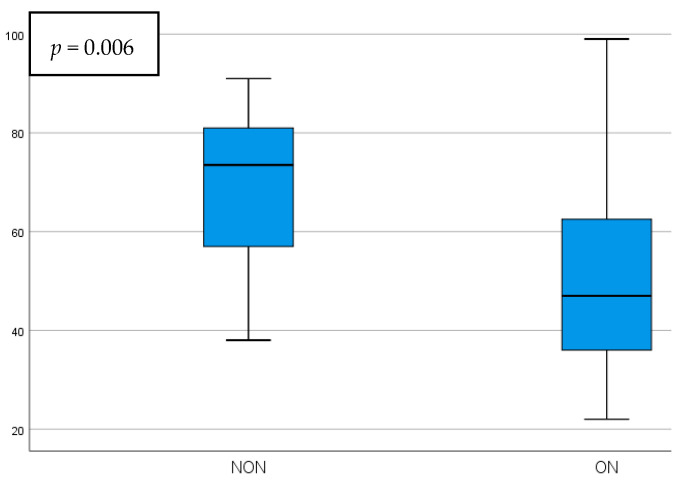
Temporal pRNFL thickness comparison in NON vs. ON eyes.

Differences between the NON and ON parameters were statistically significant when comparing the circumpapillary pRNFL thickness, temporal pRNFL thickness, and GCIPL thickness, while there were no statistically significant modifications when comparing the foveal GCIPL thickness and the p100 wave parameters. The mean values and significance can be seen in Table 2. The pRNFL thickness presented a statistically significant increase in the NON group, showing mean values of 18.27 ± 5.33 µm higher when compared to ON eyes (*p* = 0.001). The temporal pRNFL thickness had statistically significantly increased values in the NON group as well, being 15.71 ± 5.49 µm thicker than the ON group (*p* = 0.006). Schematic comparisons between the NON and ON groups regarding the circumpapillary and temporal pRNFL thickness can be seen in Figure 1 and Figure 2.

**Figure 3:**
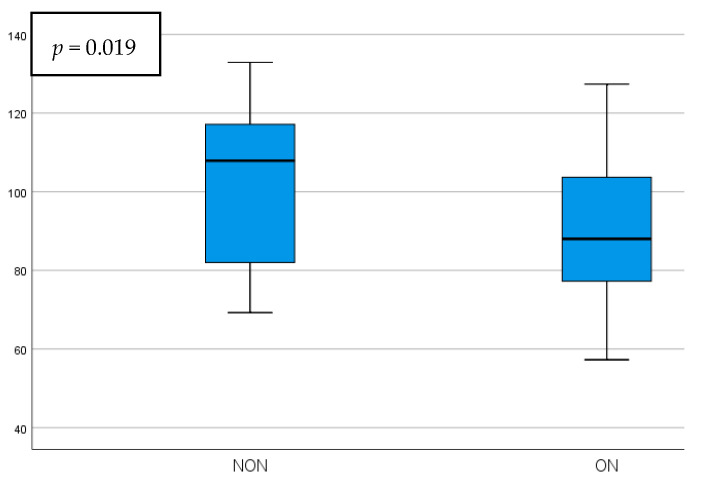
Parafoveal GCIPL thickness comparison in NON vs. ON eyes.

**Figure 4:**
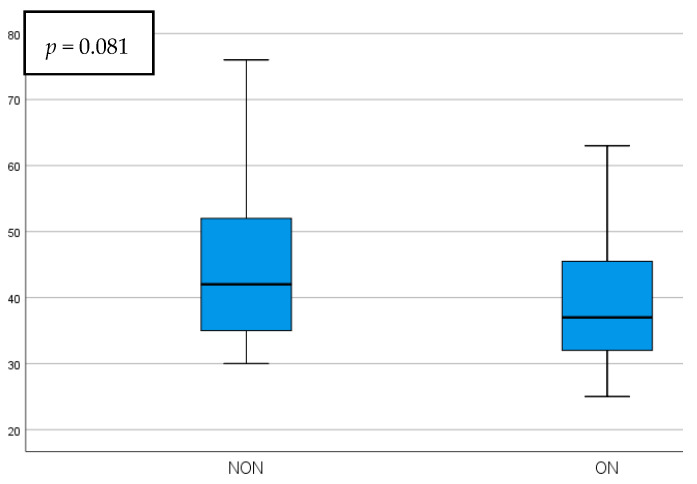
Foveal GCIPL thickness comparison in NON vs. ON eyes.

The parafoveal GCIPL thickness presented a statistically significant increase in the NON group, being 12.85 ± 5.3 µm thicker than the ON group (*p* = 0.019). While the foveal GCIPL thickness was also higher in the NON group, it did not present statistical significance. Representations of the comparisons between these parameters can be seen in Figure 3 and Figure 4.

**Figure 5:**
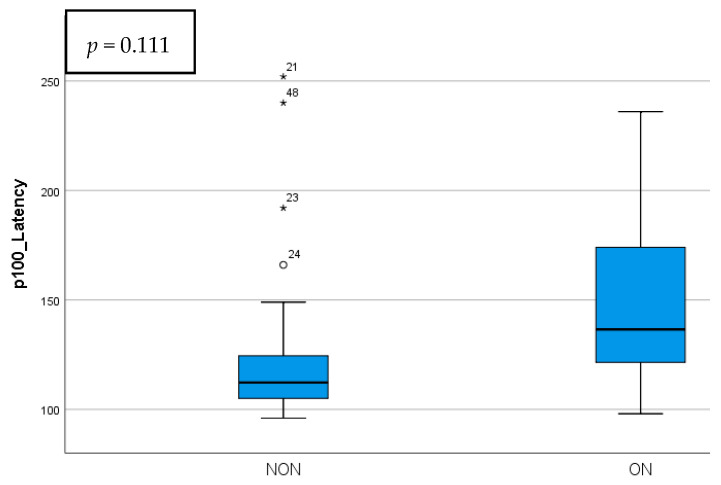
p100 wave latency comparison in NON vs. ON eyes.

**Figure 6:**
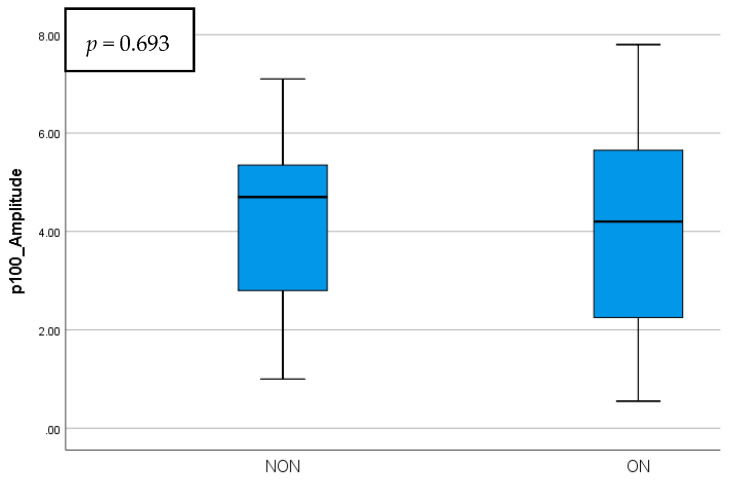
p100 wave amplitude comparison in NON vs. ON eyes.

The p100 wave parameters did not present statistical significance when taking into account the ON history. The NON group did have a shorter latency and a higher amplitude compared to the ON eyes. The differences between these parameters are presented in Figure 5 and Figure 6.

The Spearman analysis provided information regarding the correlation strength between the parameters. The NON parameters are presented in Table 3, while the ON parameters can be seen in Table 4.

In the NON group, the circumpapillary pRNFL thickness presented statistically significant, strong, positive correlations with the other OCT parameters and a negative correlation with the p100 wave latency, EDSS score, and disease duration. The ON eyes presented similar results but had also developed a positive correlation with the p100 wave amplitude.

The temporal pRNFL thickness presented similar correlations to the previous parameter. However, in the NON eyes, there was a higher value of correlation significance compared to the circumpapillary pRNFL, while the ON eyes did not present significant correlations with EDSS.

The foveal and parafoveal GCIPL thickness presented similar properties, showing statistically significant positive correlations with the optic disc parameters and negative correlations with the p100 latency and EDSS regardless of the ON history. The NON eyes showed a significant correlation with disease duration.

The p100 wavelength latency showed a statistically significant negative correlation with all the OCT parameters in both the ON and NON eyes and a positive correlation with disease duration, while only the NON eyes correlated with EDSS. While the relationship was not statistically significant, both groups presented a negative correlation with the p100 wave amplitude. The p100 wave amplitude presented a significant relationship only with the circumpapillary pRNFL thickness in ON patients; otherwise, it showed a positive correlation with the OCT parameters and a negative correlation with the latency, EDSS, and disease duration in both groups.

EDSS presented statistically significant negative correlations with the OCT parameters and positive correlations with EDSS, disease duration, and the p100 wave latency in the NON group. The ON eyes showed a similar relationship, but EDSS only correlated with the circumpapillary pRNFL thickness and the foveal and parafoveal GCIPL thickness.

The duration of the disease showed a statistically significant correlation with the other parameters in the ON eyes, whilst only with the optic disc parameters and parafoveal GCIPL thickness values and p100 wave latency in the NON eyes.

## 4. Discussion

In our study, OCT disc and parafoveal structural parameters showed a higher level of significance in differentiating between eyes with a history of ON, while the VEPs and foveal GCIPL thickness were not statistically significant. The foveal GCIPL difference could be explained by the physiologically low thickness of the GCIPL in the fovea, which is not prone to significant alterations. However, while the NON eyes had a lower value of p100 wave latency, the lack of statistical significance could be influenced by the neurodegeneration of the visual pathway caused by MS activity. In addition, the outliers in Figure 5 suggest that the small study sample could be a source of error that influenced the lack of statistical significance of this parameter. The amplitude of the p100 wave was not influenced by the ON history.

The correlations found between the temporal and circumpapillary pRNFL thickness and the other parameters, and the negative relationship of the p100 latency in the NON eyes, provide additional evidence of its capacity to be used as a biomarker capable of evaluating MS activity. The positive correlation between the circumpapillary pRNFL thickness and the p100 wave amplitude in the ON eyes offers insight regarding the lasting effects of the inflammatory episode on the optic pathway. The lack of such a correlation in the NON eyes suggests that neurodegeneration may not be solely responsible for a lower amplitude in neural transmission, but in the presence of concomitant demyelination.

The relationship between EDSS and disease activity, and the other parameters used to analyze the integrity of the optic pathway in our study, could be used to provide additional data regarding the use of optic nerve biomarkers for evaluating systemic disease activity and neurodegeneration.

The exploration of the optic pathway in MS offers multiple opportunities regarding investigative techniques. As the optic nerve is increasingly recognized as a functional extension of the CNS, there is a growing need to expand the availability and diversity of assessment methods for the optic nerve. Correlating different imaging modalities, such as fundus photography, scanning laser ophthalmoscopy, laser speckle flowgraphy, and OCT-Angiography, with established parameters for neurodegeneration of the optic nerve, such as OCT, VEPs, and MRI, will be essential for advancing our understanding of MS progression. Enhancing these diagnostic capabilities using machine learning protocols and integrating artificial intelligence could provide a more personalized approach and improve patient outcomes [16].

When comparing the capacity of discovering new clinical and subclinical ON type lesions, Grecescu found VEPs to be superior to OCT in highlighting visual function loss, whereas OCT can provide data regarding structural neurodegeneration after the inflammatory episode [17]. In a study by Di Maggio et al., VEPs were more frequently altered than OCT for NON eyes, while both presented similar sensitivity for ON eyes [18].

In our previous work, we found that while the latency of the p100 wave complex progressively increases over time, reaching statistical significance and indicating ongoing demyelination, the amplitude begins to show a significant decline only after approximately 10 years of disease duration. This temporal distinction marks a critical insight into the timing and mechanisms underlying neurodegeneration in patients with multiple sclerosis. Additionally, the presence of a negative correlation between the p100 latency and amplitude only at baseline and at later follow-up points suggests that although demyelination and neurodegeneration may have the same starting point in the early stages of MS, they follow distinct trajectories. In the advanced stages of the disease, both processes appear to be influenced by a shared neurodegenerative pattern [19].

Behbehani et al. found similar results when analyzing early diagnosed RRMS using VEPs and OCT. They found that the pRNFL thickness has a negative correlation with VEP latency, regardless of ON history, while GCIPL shows a negative correlation only in eyes with prior ON [20]. Similar to our study, they did not observe any correlations between VEP amplitude and other parameters, highlighting that axonal integrity is maintained despite neurodegeneration.

Chilińska et al. reported a negative correlation between the pRNFL thickness and the p100 latency in MS patients with an ON history. Their findings also suggest that while the amplitude of the p100 wave may recover over time, possibly reflecting remyelination, latency delays tend to persist, likely due to irreversible axonal damage and chronic neurodegeneration within the visual pathway [21].

Piedrabuena and Bittar observed a correlation between EDSS and the p100 wave latency, amplitude, and pRNFL thickness, with no statistically significant relationship to the macular volume. In addition, the authors found no difference regarding the macular volume and VEP amplitude in regard to ON history, with no significant differences in optic nerve degeneration using VEPs, OCT, and EDSS over the course of a 2-year follow-up [22].

In a similar study, Carcelén-Gadea et al. observed that over the course of 3 years, VEPs could detect optic nerve function worsening only in NON eyes, and while optic nerve damage could be objectified using multiple exploratory methods, no correlation between OCT and VEP parameters with EDSS was found [23].

The lack of progression in optic nerve atrophy in these studies could be explained by reaching a flatline level and controlling disease activity. A meta-analysis focused on longitudinal studies using OCT to evaluate pRNFL and GCIPL thickness in MS observed a significant thinning rate over time [24]. While Balk et al. provide data regarding important degeneration in early MS progression with a decrease in the pRNFL and GCIPL thickness of >1,1 μm/year in the first 2 years of disease activity, the authors consider that a longer disease duration will generate atrophy attenuation in a plateau aspect [25]. Bsteh et al. also used the annual atrophy of the retinal layers to demonstrate treatment failure in RRMS [26]. In another paper, the authors propose establishing a new baseline when starting disease-modifying therapies in order to better highlight current disease activity [27].

Large cohort studies assessed pRNFL and GCIPL thickness and compared them to MRI for detecting neurodegeneration. While they found that disease activity did not correlate with variations in OCT parameters, they found a strong correlation between the GCIPL thickness and the cerebral volume measured through MRI, proposing the measurement of GCIPL for evaluating disease activity and neurodegeneration [28,29].

In a multicentric study, Oertel et al. evaluated the ability of the p100 wave latency obtained using VEPs to predict axonal loss over the anterior visual pathway. Their findings suggest that the parameter could be used to evaluate the GCIPL atrophy rate. Furthermore, since the GCIPL is an established predictor of disease disability level, VEPs could be used to predict long-term disability in the RRMS population [30].

Pihl-Jensen et al. evaluated 79 clinically isolated syndrome patients with ON, out of which 28 later converted to MS. They found that the GCIPL and pRNFL thickness predicted the development of MS in a multivariate analysis, while multifocal VEP latency could only predict it in a univariate analysis, highlighting the properties of these parameters to act as biomarkers [31].

When analyzing conversion to secondary progressive MS, Eklund et al. found the OCT-measured GCIPL thickness and VEP-analyzed p100 latency to be correlated with EDSS and useful in monitoring disease progression, providing support for analyzing different MS subtypes using these investigations [32].

Recurrent demyelination in the same optic nerve region can disrupt natural repair mechanisms, leading to permanently demyelinated axons and remyelination failure. This could offer insight into why remyelination occurs early in MS but is often absent in chronic lesions with repeated damage. Although beneficial, remyelination has limitations, resulting in thinner myelin, shorter internodes, and reduced nerve transmission, contributing to persistent visual deficits after ON [33].

Given the rising prevalence of vision-related impairments affecting quality of life in children and the non-invasive nature of these assessment methods [34], OCT and VEPs could be used to evaluate MS activity in the pediatric population [35]. OCT could be used to provide baseline values that patients will require later, regardless of ON history, in order to evaluate neurodegeneration, especially due to longer disease activity. Kothari et al. found that there is increased reproducibility of VEPs in school children aged 7–12, confirming their use for evaluating optic path integrity in the pediatric population [36]. Nikolic et al. found that ON is a commonly occurring clinical manifestation in pediatric MS patients, with 40% of patients presenting subclinical VEP alterations and parameters being correlated with residual visual defects [37].

While pregnancy is associated with decreased rates in MS progression and patients interrupt treatment over the course of gestation, the mechanisms are not fully understood. In combining structural and vascular parameters using OCT and OCT-Angiography, studies have provided evidence of variation in retinal circulation based on sex hormone levels [38,39,40]. Therefore, OCT and VEPs could be used to understand the way hormone levels influence disease activity [41].

With the increasing evidence of the capacity of OCT and OCT-Angiography parameters to differentiate between different demyelinating CNS diseases, correlations with VEPs may provide additional evidence regarding the pathophysiological mechanism, guiding towards faster diagnosis and treatment in these patients [42,43,44,45].

Serial VEP assessments have shown that corticosteroid therapy administered during inflammatory episodes can lead to improvements in electrophysiological responses. The normalization of VEP patterns following treatment is indicative of inflammatory remission and may suggest the preservation of axonal integrity, supporting a more favorable visual and neurological prognosis [46].

The limitations of our study consist of the low number of participants and a lack of longitudinal data to support our findings over time. In addition, the studied population is heterogeneous regarding age and disease duration, and while this may add value regarding the validity of our results in the MS population, future studies should focus on reproducing these aspects, taking into account different stages in MS progression and other physiological aspects that could influence the results. Furthermore, some patients could have a history of multiple ON episodes, which could lead to additional sources of error when interpreting the results, and larger studies should include this element in their analysis.

Future directions should focus on integrating AI-based machine learning models in order to search for specific correlations between OCT and VEP alterations and the MRI lesion site. In addition, analyzing vascular parameters using OCT-Angiography could provide additional data regarding disease activity, with studies showing that VEP measurements have a correlation with the optic nerve head and the circumpapillary vascular density. Such data may provide additional evidence of the lasting structural damage after an inflammatory episode, which could cause alterations in optic nerve blood flow [47]. Moreover, by including progressive MS subtypes in a larger cohort study, data regarding the presence of our findings across the MS spectrum could be obtained.

## 5. Conclusions

Our findings highlight the value of combining OCT and VEPs as complementary tools for evaluating structural and functional changes in the visual pathway of MS patients. Eyes without a history of ON demonstrated significantly thicker pRNFL and parafoveal GCIPL layers compared to ON eyes, emphasizing the structural impact of inflammatory episodes. Although the VEP parameters did not differ significantly between the groups, their correlation with the OCT measurements, EDSS, and disease duration supports their relevance in assessing demyelination and neurodegeneration.

## Figures and Tables

**Table 1:** Descriptive parameters.

Parameters	NON Mean ± SD	NON Median ± SD	ON Mean ± SD	ON Median ± SD
pRNFL T	104.24 ± 15.84 µm	108.5 ± 15.84 µm	85.98 ± 21.21 µm	83.63 ± 17.61 µm
pRNFL Temporal T	67.95 ± 17.61 µm	73.5 ± 17.61 µm	52.25 ± 21.15 µm	47 ± 21.15 µm
Parafoveal GCIPL T	102.69 ± 19.29 µm	107.88 ± 19.29 µm	89.85 ± 19.03 µm	88 ± 19.03 µm
Foveal GCIPL T	45.86 ± 13.54 µm	42 ± 13.54 µm	39.97 ± 10.75 µm	37 ± 10.75 µm
p100 Latency	128.75 ± 44.68 ms	112.25 ± 44.68 ms	145.69 ± 32.09 ms	136.5 ± 32.09 ms
p100 Amplitude	4.33 ± 1.7 mV	4.7 ± 1.7 mV	4.12 ± 2.1 mV	4.2 ± 2.1 mV

**Table 2:** Independent sample *t*-test between NON and ON eyes.

Parameters	Mean Difference ± SD	*p*
pRNFL T	18.27 ± 5.33 µm	0.001
pRNFL Temporal T	15.71 ± 5.49 µm	0.006
Parafoveal GCIPL T	12.85 ± 5.3 µm	0.019
Foveal GCIPL T	5.9 ± 3.31 µm	0.081
p100 Latency	−16.93 ± 10.44 ms	0.111
p100 Amplitude	0.21 ± 0.53 mV	0.693

**Table 3:** Spearman correlation between analyzed parameters in NON MS patients.

Parameters		pRNFL T	pRNFL Temporal T	Parafoveal GCIPL T	Foveal GCIPL T	p100 Latency	p100 Amplitude	EDSS	Disease Duration
pRNFL T	r	1.000	0.805 **	0.890 **	0.635 **	−0.699 **	0.222	−0.496 *	−0.537 *
	*p*	.	**0.000**	**0.000**	**0.002**	**0.000**	0.322	**0.019**	**0.010**
pRNFL Temporal T	r	0.805 **	1.000	0.795 **	0.687 **	−0.671 **	0.118	−0.556 **	−0.577 **
	*p*	**0.000**	.	**0.000**	**0.000**	**0.001**	0.601	**0.007**	**0.005**
Parafoveal GCIPL T	r	0.890 **	0.795 **	1.000	0.574 **	−0.622 **	−0.086	−0.587 **	−0.518 *
	*p*	**0.000**	**0.000**	.	**0.005**	**0.002**	0.702	**0.004**	**0.014**
Foveal GCIPL T	r	0.635 **	0.687 **	0.574 **	1.000	−0.502 *	0.061	−0.594 **	−0.577 **
	*p*	**0.002**	**0.000**	**0.005**	.	**0.017**	0.788	**0.004**	**0.005**
p100 Latency	r	−0.699 **	−0.671 **	−0.622 **	−0.502 *	1.000	−0.155	0.553 **	0.491 *
	*p*	**0.000**	**0.001**	**0.002**	**0.017**	.	0.492	**0.008**	**0.020**
p100 Amplitude	r	0.222	0.118	−0.086	0.061	−0.155	1.000	0.324	0.035
	*p*	0.322	0.601	0.702	0.788	0.492	.	0.141	0.878
EDSS	r	−0.496 *	−0.556 **	−0.587 **	−0.594 **	0.553 **	0.324	1.000	0.753 **
	*p*	**0.019**	**0.007**	**0.004**	**0.004**	**0.008**	0.141	.	**0.000**
Disease Duration	r	−0.537 *	−0.577 **	−0.518 *	−0.577 **	0.491 *	0.035	0.753 **	1.000
	*p*	**0.010**	**0.005**	**0.014**	**0.005**	**0.020**	0.878	**0.000**	.

**. Correlation is significant at the 0.01 level (two-tailed). *. Correlation is significant at the 0.05 level (two-tailed). Bold has been used to highlight statistically significant values.

**Table 4:** Spearman correlation between analyzed parameters in ON MS patients.

Parameters		pRNFL T	pRNFL Temporal T	Parafoveal GCIPL T	Foveal GCIPL T	p100 Latency	p100 Amplitude	EDSS	Disease Duration
pRNFL T	r	1.000	0.903 **	0.891 **	0.646 **	−0.583 **	0.351 *	−0.410 *	−0.430 *
	*p*	.	**0.000**	**0.000**	**0.000**	**0.000**	**0.049**	**0.020**	**0.014**
pRNFL Temporal T	r	0.903 **	1.000	0.955 **	0.732 **	−0.600 **	0.341	−0.330	−0.398 *
	*p*	**0.000**	.	**0.000**	**0.000**	**0.000**	0.056	0.065	**0.024**
Parafoveal GCIPL T	r	0.891 **	0.955 **	1.000	0.744 **	−0.601 **	0.326	−0.423 *	−0.398 *
	*p*	**0.000**	**0.000**	.	**0.000**	**0.000**	0.068	**0.016**	**0.024**
Foveal GCIPL T	r	0.646 **	0.732 **	0.744 **	1.000	−0.406 *	0.047	−0.378 *	−0.149
	*p*	**0.000**	**0.000**	**0.000**	.	**0.021**	0.799	**0.033**	0.415
p100 Latency	r	−0.583 **	−0.600 **	−0.601 **	−0.406 *	1.000	−0.188	0.297	0.446 *
	*p*	**0.000**	**0.000**	**0.000**	**0.021**	.	0.303	0.099	**0.010**
p100 Amplitude	r	0.351 *	0.341	0.326	0.047	−0.188	1.000	−0.217	0.131
	*p*	**0.049**	0.056	0.068	0.799	0.303	.	0.234	0.473
EDSS	r	−0.410 *	−0.330	−0.423 *	−0.378 *	0.297	−0.217	1.000	0.294
	*p*	**0.020**	0.065	**0.016**	**0.033**	0.099	0.234	.	0.103
Disease Duration	r	−0.430 *	−0.398 *	−0.398 *	−0.149	0.446 *	0.131	0.294	1.000
	*p*	**0.014**	**0.024**	**0.024**	0.415	**0.010**	0.473	0.103	.

**. Correlation is significant at the 0.01 level (two-tailed) *. Correlation is significant at the 0.05 level (two-tailed). Bold has been used to highlight statistically significant values.

## Data Availability

The original contributions presented in this study are included in the article. Further inquiries can be directed to the corresponding author.
